# Genetic aberrations detected by comparative genomic hybridisation in vulvar cancers

**DOI:** 10.1038/sj.bjc.6600112

**Published:** 2002-03-18

**Authors:** D G Allen, A-M Hutchins, F Hammet, D J White, J P Scurry, S N Tabrizi, S M Garland, J E Armes

**Affiliations:** Department of Gynecologic Oncology, Mercy Hospital for Women, Melbourne, Australia; Department of Pathology, Mercy Hospital for Women, Melbourne, Australia; Molecular Pathology Laboratory, Victorian Breast Cancer Research Consortium, University of Melbourne and Peter MacCallum Cancer Institute, Melbourne, Australia; Department of Pathology, University of Melbourne and Peter MacCallum Cancer Institute, Melbourne, Australia; Department of Microbiology and Infectious Diseases, Royal Women's Hospital, Women's and Children's Health, Melbourne, Australia; Department of Anatomical Pathology, Royal Women's Hospital, Women's and Children's Health, Melbourne, Australia

**Keywords:** comparative genomic hybridisation, vulva, neoplasm, squamous cell carcinoma, human papillomavirus

## Abstract

Squamous cell carcinoma of the vulva is a disease of significant clinical importance, which arises in the presence or absence of human papillomavirus. We used comparative genomic hybridisation to document non-random chromosomal gains and losses within human papillomavirus positive and negative vulvar cancers. Gain of 3q was significantly more common in human papillomavirus-positive cancers compared to human papillomavirus-negative cancers. The smallest area of gain was 3q22–25, a chromosome region which is frequently gained in other human papillomavirus-related cancers. Chromosome 8q was more commonly gained in human papillomavirus-negative compared to human papillomavirus-positive cancers. 8q21 was the smallest region of gain, which has been identified in other, non-human papillomavirus-related cancers. Chromosome arms 3p and 11q were lost in both categories of vulvar cancer. This study has demonstrated chromosome locations important in the development of vulvar squamous cell carcinoma. Additionally, taken together with previous studies of human papillomavirus-positive cancers of other anogenital sites, the data indicate that one or more oncogenes important in the development and progression of human papillomavirus-induced carcinomas are located on 3q. The different genetic changes seen in human papillomavirus-positive and negative vulvar squamous cell carcinomas support the clinicopathological data indicating that these are different cancer types.

*British Journal of Cancer* (2002) **86**, 924–928. DOI: 10.1038/sj/bjc/6600112 www.bjcancer.com

© 2002 Cancer Research UK

## 

Vulvar cancers affect 1.6 per 100 000 women in Victoria, Australia, 85% of which are squamous cell carcinomas (SCC) (Canstat, 1998). There are two clinicopathological types of SCC of the vulva: those that contain human papillomavirus (HPV) DNA and those that do not. HPV-positive vulvar SCCs share common epidemiological features with SCC of the vagina, cervix and anal region. HPV has been detected in less than 50% of vulvar SCCs (in Frierson and Mills, 1999). These HPV-positive tumours occur in women on average 14 years younger when compared to HPV-negative vulvar cancers, tend to be basaloid in appearance and arise from classical (Bowenoid) vulvar intraepithelial neoplasia (VIN) (Scurry *et al*, 1998). The pathogenesis of HPV-negative cancers is less well understood, but these cancers frequently arise on a background of lichen sclerosus, squamous cell hyperplasia and simplex (differentiated) type of VIN. These different clinicopathological features suggest that there may be different genetic changes in HPV-positive and negative cancers.

The process of HPV-related carcinogenesis within SCC of the anogenital region depends on functions encoded by the viral E6 and E7 genes. These genes bind with the p53 and Rb genes, respectively, inactivating their function in cell cycle regulation (Munger *et al*, 1989). However, many women have been exposed to HPV without developing premalignant or malignant changes within the vulva, and thus it is likely that additional genetic changes are necessary for tumour development and progression. Furthermore, other than a small number of studies addressing loss of heterozygosity using a limited number of markers, few studies have addressed whether HPV-positive vulvar cancers share similar genetic changes to those seen in HPV-negative cancers, or whether these two types of SCC of the vulva share separate molecular pathogeneses (Flowers *et al*, 1999; Pinto *et al*, 1999; Scurry *et al*, 1999).

Comparative genomic hybridization (CGH) is a powerful tool in the study of cancer that has fast gained recognition for its ability to scan an entire genome for DNA copy number changes that are mapped to chromosome regions. HPV-positive and negative vulvar cancers were analysed using CGH in order to determine specific chromosomal regions of DNA copy number gain and loss and to compare these changes with the HPV status of the tumours.

## PATIENTS AND METHODS

The 18 patients with vulvar cancer used in this study were treated surgically between 1982 and 1999 at The Mercy Hospital for Women. Pathologic review was performed and the histologic type confirmed as SCC.

### CGH methods

CGH was carried out according to methods described in detail elsewhere (Allen *et al*, 2000). Briefly, areas of interest (2–3 mm^2^) were microdissected from paraffin-embedded formalin-fixed tissue sections and digested with proteinase K. MCF7 cell-line DNA and reference control DNAs from peripheral blood lymphocytes were extracted using a Progenome kit (Progen, Ipswich, Australia). DOP–PCR was performed on all reference and tumour samples and MCF-7 control DNA as previously described by Kuukasjarvi *et al* (1997). The CGH result using DOP–PCR amplified MCF-7 control showed all the same changes as an intra-laboratory MCF-7 standard CGH profile using non-amplified DNA. Amplified DNA samples were labelled by nick translation using the Bionick labelling system (Life Technologies, Gaithersburg, MD, USA). Both a primary and confirmatory CGH experiment was performed for each sample using different labels to control for variation of different fluorochromes. Firstly, tumour samples were directly labelled with Spectrum Red and hybridised with biotin labelled reference DNA. In the confirmatory hybridisation tumour samples were labelled with digoxigenin and hybridised with reference DNA directly labelled with Spectrum Green. Only changes seen in both hybridisations were used in the results. Following washing, biotinylated DNA was detected by streptavidin-conjugated fluorescein isothiocyanate (FITC) (Vector Laboratories, Burlingame, CA, USA) and digoxigenin-labelled DNA by mouse anti-digoxigenin antibody conjugated to rhodamine (TRITC) (Boehringer-Mannheim, Germany). Samples were counterstained with 4,6-diamino-2-phenylindole (DAPI). Each CGH experiment included at least one normal lymph node DNA as a negative control. Images were analysed with Quips CGH Analysis Software (Vysis Inc, Downers Grove, IL, USA). A gain of DNA sequence copy number was defined by a tumour/reference ratio >1.2 on both standard and inverse hybridisation. A copy number decrease was defined as having a tumour/reference ratio below 0.8 on both hybridisations. No distinction was attempted between high-level copy number increases (amplifications) of subregions as contrasted to gains of a whole arm.

### HPV methods

Determination of HPV status was carried out according to methods described in detail elsewhere (Allen *et al*, 2000). Briefly 7 μm sections of paraffin-embedded vulvar tumours were dewaxed, and digested with proteinase. Samples were amplified for 40 cycles using 50 ρmol of each of the Ll consensus primers MY09 and MY11 (Manos *et al*, 1989) and 5 ρmol of each of the two beta-globin primers GH20-PC04 (Resnick *et al*, 1990). DNA from cloned HPV 6, 11, 16, 18, 31 and 33 was used as positive controls and DNA extracted from normal placenta as negative controls. Amplification products were hybridised with biotin-labelled HPV Ll generic probe (Bauer *et al*, 1991) and captured on streptavidin coated plates (Roche Biochemicals) (Layton-Henry *et al*, 1996). The bound hybrid was detected by an anti-digoxigenin peroxidase conjugate by use of the colourimetic substrate ABTS (Layton-Henry *et al*, 1996). The positive HPV specimens were typed for HPV 6, 11, 16, 18, 31, 33, 35, 39, 45, 51, 52 with type-specific probes (Resnick *et al*, 1990) using similar detection methods as described above.

### Statistical analysis

The Fisher's exact test was used to compare proportions from two independent samples, while the two-sample independent-groups *t*-test was used to compare the difference between means. All tests were two-sided and results with a *P*-value of less than 0.05 were considered statistically significant.

## RESULTS

**Figure 1:**
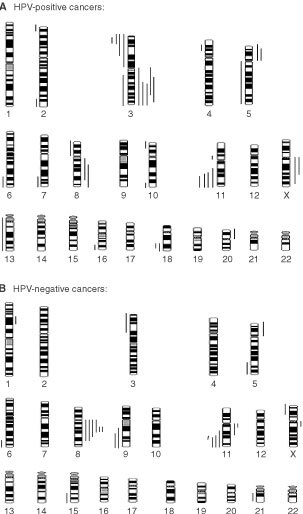
Chromosome gains and losses in vulvar SCCs. (**A**) HPV-positive cancers and (**B**) HPV-negative cancers. Bars on the left side of the idiograms indicate a loss and bars on the right side indicate a gain of genetic material.

The HPV analysis showed that there were 8 HPV-negative tumours and 10 HPV-positive tumours. All HPV-positive tumours contained subtype 16. Figure 1

represents the overall gains and losses in the 18 vulvar tumours, mapped to specific chromosome locations. The mean number of overall changes seen in the 18 tumours was 3.2 per case (range 0–5). This comprised a mean of 1.3 gains per case (range 0–3) and a mean of 1.9 losses per case (range 0–5). There were more CGH changes in the HPV-positive group, compared to the HPV-negative group (mean 3.6, *vs* 2.8, respectively) but this did not reach statistical significance. The increased number of changes in the HPV-positive group was due to an excess number of losses in these tumours (mean number of losses 2.4, *vs* 1.4, respectively). HPV-positive and negative cancers had a mean of 1.2 and 1.4 gains per case, respectively.

Table 1

**Table 1:**
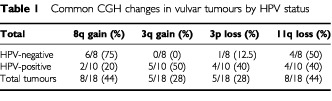
Common CGH changes in vulvar tumours by HPV status

shows the four most common CGH changes in the vulvar cancers and Figure 2

**Figure 2:**
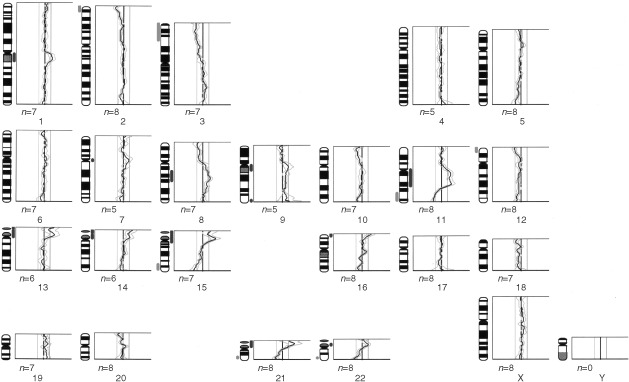
An individual CGH ratio profile of a representative case of HPV-negative vulvar cancer. Gains are represented to the right of the midline on the ideogram and losses to the left.

shows a representative CGH profile on an individual case. Chromosomal locations 8q (8 out of 18, 44%) and 3q (5 out of 18, 28%) were most frequently affected by genetic gain, whilst 11q (8 out of 18, 44%) and 3p (5 out of 18, 28%) were most frequently lost. There was a statistically significant difference at the *P*=0.05 level, in 3q and 8q gain when analysed by HPV status, with 3q gain being more common in HPV-positive cancers (5 out of 10 *vs* 0 out of 8, *P*<0.025), and 8q gain more common in HPV-negative cancers (6 out of 8 *vs* 2 out of 10, *P*<0.025). There was no statistically significant difference by HPV status for 11q and 3p loss. Whilst many tumours showed whole chromosome arm alteration, minimal regions of gain and loss could be determined from some individual tumours. Thus the smallest areas of copy number gain were mapped to 3q22–25 and 8q21 and loss to 3p24, and 11q22-qter.

## DISCUSSION

In this study we have performed CGH analysis of SCC of the vulva, with known HPV status. To our knowledge, this is the first report of a CGH-based, genome-wide analysis of chromosomal gains and losses on this cancer type analysed by HPV status. Vulvar cancer differs from other SCCs of the anogenital region, since a substantial proportion of vulvar cancers are HPV-negative. The different HPV status of these cancers is reflected in their different clinicopathological parameters. We have utilised CGH to identify chromosomal loci important in vulvar oncogenesis in general, as well as those which differ according to the HPV status.

Several regions of recurrent chromosomal abnormality were identified in vulvar SCCs. Notably gain of chromosome 3q was a frequent finding in HPV-positive SCCs (50%), but not detected in any of our HPV-negative cases. These data support gain of 3q as an important event in the oncogenesis of HPV-related SCCs since, in a previous study, we have shown that a similar proportion of stage 1b HPV-positive cervical SCCs harbour 3q gain (Allen *et al*, 2000). Gain of 3q has also been detected by others in cervical SCCs (Heselmeyer *et al*, 1996, 1997b; Dellas *et al*, 1999; Kirchoff *et al*, 1999) and in HPV-positive SCC of the anus and its precursor lesions (Heselmeyer *et al*, 1997a; Haga *et al*, 2001). Further, we were able to map the smallest region of gain in vulvar SCCs to 3q22–25, whilst our previous series of cervical cancers showed the smallest area of gain to be 3q24–26 (Allen *et al*, 2000). Only one previous CGH analysis of vulvar carcinoma has been published, which detected 3q gain as the most common region of gain (40% of 10 cases) (Jee *et al*, 2001). However, these tumours were not assessed for HPV status. Our data indicate that 3q gain is important primarily in HPV-positive vulvar cancers. Taken together, our present data and past findings on cervical SCC, as well as previous data showing 3q gain in other HPV-related anogenital SCCs, suggest that this region of the genome harbours gene(s) synergistic with HPV in oncogenesis and which are important in the progression of HPV-induced SCCs of the anogenital region.

Loss of the chromosome arms 11q and 3p were also frequently detected in our series of vulvar cancers. Chromosome 3p loss has been previously documented in vulvar SCCs by LOH studies (Flowers *et al*, 1999; Pinto *et al*, 1999) and by CGH (Jee *et al*, 2001). The previous LOH studies indicate that the loss of 3p markers was not related to HPV status, which is supported by our own CGH data. Furthermore, two of the microsatellite markers showing LOH which were used in the previous studies were mapped to the region of 3p24, which was the smallest region of chromosome loss detected by CGH in the current study. Loss of 3p is also frequently seen in cervical SCC, by CGH and LOH analysis (Heselmeyer *et al*, 1996, 1997b; Larson *et al*, 1997; Wistuba *et al*, 1997; Kersemaekers *et al*, 1998; Steenbergen *et al*, 1998; Dellas *et al*, 1999; Kirchoff *et al*, 1999; Allen *et al*, 2000). Loss of 11q has been documented in cervical and other malignancies not related to HPV infection, including breast, colorectal and ovarian cancers and malignant melanoma (Hampton *et al*, 1994; Kersemaekers *et al*, 1998; Allen *et al*, 2000). Loss of 11q was frequently documented in our series of vulvar cancers and was not related to HPV status. The smallest region of loss could be mapped to 11q22-qter. We have previously mapped the smallest region of chromosome loss on 11q in cervical cancers as 11q22–24 (Allen *et al*, 2000) and an identical region has been previously mapped by LOH studies in cervical cancer (Hampton *et al*, 1994). These data indicate that, in contrast to genes on 3q, those on 3p and 11q may be related to development and progression of anogenital SCC independent of HPV status.

Recurrent gain of 8q was also detected in our series of vulvar cancers. Interestingly, this gain was much more frequent in HPV-negative compared to HPV-positive cancers (75% *vs* 20%, respectively). The smallest area of gain was 8q21, which is centromeric to the location of the proto-oncogene *MYC*. Gain of 8q, including the 8q21 region, has been previously documented in many different cancer types, the majority of which are not related to HPV infection (Kallioniemi *et al*, 1994; Prat *et al*, 2001; Shiraishi *et al*, 2001; van Dekken *et al*, 2001). The current and other studies therefore support the notion that 8q houses important oncogene(s) other than *MYC*. Although our data would suggest that 8q gain is important in the development of HPV-negative vulvar cancer, the number of cases in the present study are too small to clearly indicate whether 8q gain is redundant in the development of HPV-positive vulvar cancers, as is documented for mutations in the p53 gene (Flowers *et al*, 1999; Brooks *et al*, 2000).

Only one previous study describing CGH analysis of vulvar carcinomas has been published (Jee *et al*, 2001). However, the HPV status of these cases was unreported. Whilst the majority of changes detected in this previous series is similar to our own, Jee *et al* (2001) found the commonest detectable change to be 4p loss (5 out of 10 cases). 4p loss was only detected in one of our 18 cases (an HPV-positive case). Interestingly, one previous study of regions of genomic loss in vulvar cancer detected by LOH found only two of 11 cases to show loss of the 4p marker D4S2366 (Pinto *et al*, 1999). It is possible therefore that the difference in detection of this regional loss between our own study and that of Jee *et al* (2001) is due to the different demographics of the two populations studied, confounded by the small number of cases studied in each series and the probable relatively low incidence of 4p loss in vulvar cancers overall.

Our findings indicate that SCC of the vulva harbour several chromosomal alterations in common with other SCCs of the anogenital region. Additionally, there appear to be selected chromosomal regions in which copy number abnormalities are related to HPV status. Identification of candidate genes in these areas of recurrent chromosomal copy number abnormality would give important insights into the development and progression of vulvar and other anogenital cancers.

## References

[bib1] AllenDGWhiteDJHutchinsA-MScurryJPTabriziSNGarlandSMArmesJE2000 Progressive genetic aberrations detected by comparative genomic hybridisation in squamous cell cervical cancers Br J Cancer 83 16591663 11104563 10.1054/bjoc.2000.1509 PMC2363460

[bib2] BauerHMTingYGreerCEChambersJCTashiroCJChimeraJReingoldAManosMM1991 Genital human papillomavirus infection in female university students as determined by a PCR-based method JAMA 265 472474 1845912

[bib3] BrooksLATidyJAGustersonBHillerLO'NionsJGascoMMarinMCFarrellPJKaelinWGJRCrookT2000 Preferential retention of codon 72 arginine p53 in squamous cell carcinomas of the vulva occurs in cancers positive and negative for human papillomavirus Cancer Res 60 68756877 11156383

[bib4] Canstat1998 Cancer in Victoria 32 19

[bib5] DellasATorhorstJJiangFProffittJSchultheissEHolzgreveWSauterGMihatschMJMochH1999 Prognostic value of genomic alterations in invasive cervical squamous cell carcinoma of clinical stage IB detected by comparative genomic hybridization Cancer Res 59 34753479 10416613

[bib6] FlowersLCWistubaIIScurryJMullerCYAshfaqRMillerDSMinnaJDGazdarAF1999 Genetic changes during the multistage pathogenesis of human papillomavirus positive and negative vulvar carcinomas J Soc Gynecol Investig 6 213221 10.1016/s1071-5576(99)00023-4 10486784

[bib7] FriersonHRMillsSE1999 The vulva and vaginaInDiagnostic Surgical PathologySternberg SS (ed)pp21112153Philadelphia: Lippincott Williams and Wilkins

[bib8] HagaTKimSHJensenRHDarraghTPalefskyJM2001 Detection of genetic changes in anal intraepithelial neoplasia (AIN) of HIV-positive and HIV-negative men J Acquir Immune Defic Syndr 26 256262 11242198 10.1097/00042560-200103010-00007

[bib9] HamptonGMPennyLABaergenRNLarsonABrewerCLiaoSBusby-EarleRMWilliamsAWSteelCMBirdCCStanbridgeEJEvansGA1994 Loss of heterozygosity in cervical carcinoma: subchromosomal localization of a putative tumour-suppressor gene to chromosome 11q22-q24 Proc Natl Acad Sci USA 91 69536957 8041728 10.1073/pnas.91.15.6953 PMC44316

[bib10] HeselmeyerKdu ManoirSBlegenHFribergBSvenssonCSchrockEVeldmanTShahKAuerGRiedT1997a A recurrent pattern of chromosomal aberrations and immunophenotypic appearance defines anal squamous cell carcinomas Br J Cancer 76 12711278 9374370 10.1038/bjc.1997.547 PMC2228157

[bib11] HeselmeyerKMacvilleMSchrockEBlegenHHellstromAShahKet al1997b Advanced-stage cervical carcinomas are defined by a recurrent pattern of chromosomal aberrations revealing high genetic instability and a consistent gain of chromosomal arm 3q Genes Chromosomes Cancer 19 233240 9258658

[bib12] HeselmeyerKSchrockEDu ManoirSBlegenHShahKSteinbeckRet al1996 Gain of chromosome 3q defines the transition from severe dysplasia to invasive carcinoma of the uterine cervix Proc Natl Acad Sci USA 93 479484 8552665 10.1073/pnas.93.1.479 PMC40262

[bib13] JeeKJKimYTKimKRKimHSYanAKnuutilaS2001 Loss in 3p and 4p and gain of 3q are concomitant aberrations in squamous cell carcinoma of the vulva Mod Pathol 14 377381 11353045 10.1038/modpathol.3880321

[bib14] KallioniemiAKallioniemiOPPiperJTannerMStokkeTChenLSmithHSPinkelDGrayJWWaldmanFM1994 Detection and mapping of amplified DNA sequences in breast cancer by comparative genomic hybridization Proc Natl Acad Sci USA 91 21562160 8134364 10.1073/pnas.91.6.2156 PMC43329

[bib15] KersemaekersAMHermansJFleurenGJvan de VijverMJ1998 Loss of heterozygosity for defined regions on chromosomes 3, 11 and 17 in carcinomas of the uterine cervix Br J Cancer 77 192200 9460988 10.1038/bjc.1998.33 PMC2151237

[bib16] KirchoffMRoseHPetersonBLet al1999 Comparative genomic hybridization reveals a recurrent pattern of chromosomal aberrations in severe dysplasia/carcinoma in situ of the cervix and in advanced-stage cervical carcinoma Genes Chromos Cancer 24 144150 9885981 10.1002/(sici)1098-2264(199902)24:2<144::aid-gcc7>3.0.co;2-9

[bib17] KuukasjarviTTannerMPennanenSKarhuRVisakorpiTIsolaJ1997 Optimizing DOP-PCR for universal amplification of small DNA samples in comparative genomic hybridization Genes Chromosomes Cancer 18 94101 9115969

[bib18] LarsonAALiaoSYStanbridgeEJCaveneeWKHamptonGM1997 Genetic alterations accumulate during cervical tumorigenesis and indicate a common origin for multifocal lesions Cancer Res 57 41714176 9331069

[bib19] Layton-HenryJScurryJSPlannerRSAllenDSykesPGarlandSMBorgAJTabriziSN1996 Cervical adenoid basal carcinoma, five cases and literature review Int J Gynecol Cancer 6 193199

[bib20] ManosMMTingYWrightDKLewisAJBrokerTRWolinskySM1989 The use of polymerase chain reaction amplification for the detection of genital human papillomaviruses Cancer Cells 7 209214

[bib21] MungerKPhelpsWCBubbVHowleyPMSchlegalR1989 The E6 and E7 genes of the human papillomavirus type 16 together are necessary and sufficient for transformation of human primary keratinocytes J Virol 63 44174421 2476573 10.1128/jvi.63.10.4417-4421.1989 PMC251060

[bib22] PintoAPLinMCMutterGLSunDVillaLLCrumCP1999 Allelic loss in human papillomavirus-positive and -negative vulvar squamous cell carcinomas Am J Pathol 154 10091015 10233839 10.1016/S0002-9440(10)65353-9 PMC1866571

[bib23] PratEBernuesMCaballinMREgozcueJGelabertAMiroR2001 Detection of chromosomal imbalances in papillary bladder tumors by comparative genomic hybridization Urology 57 986992 11337313 10.1016/s0090-4295(01)00909-8

[bib24] ResnickRMCornelissenMTWrightDKet al1990 Detection and typing of human papillomavirus in archival cervical cancer specimens by DNA amplification with consensus primers J Natl Cancer Inst 82 14771484 2167986 10.1093/jnci/82.18.1477

[bib25] ScurryJFlowersLWistubaIVaninKMulvanyNReyesHGazdarA1998 Human papillomavirus, lichen sclerosis and vulvar squamous cell carcinoma Int J Gynecol Cancer 8 298306

[bib26] ScurryJHungJFlowersLKneafsayPGazdarA1999 Ploidy in human papillomavirus positive and negative vulvar squamous cell carcinomas and adjacent skin lesions Int J Gynecol Cancer 9 187193 11240765 10.1046/j.1525-1438.1999.99015.x

[bib27] ShiraishiKOkitaKKusanoNHaradaTKondohSOkitaSRyozawaSOhmuraRNoguchiTIidaYAkiyamaTOgaAFukumotoYFuruyaTKawauchiSSasakiK2001 A comparison of DNA copy number changes detected by comparative genomic hybridization in malignancies of the liver, biliary tract and pancreas Oncology 60 151161 11244331 10.1159/000055313

[bib28] SteenbergenRDHermsenMAWalboomersJMMeijerGABaakJPMeijerCJSnijdersPJ1998 Non-random allelic losses at 3p, 11p and 13q during HPV-mediated immortalization and concomitant loss of terminal differentiation of human keratinocytes Int J Cancer 76 412417 9579580 10.1002/(sici)1097-0215(19980504)76:3<412::aid-ijc20>3.0.co;2-b

[bib29] van DekkenHAlersJCRiegmanPHRosenbergCTilanusHWVissersK2001 Molecular cytogenetic evaluation of gastric cardia adenocarcinoma and precursor lesions Am J Pathol 58 19611967 10.1016/S0002-9440(10)64666-4 PMC1891976 11395372

[bib30] WistubaIIMontellanoFDMilchgrubSVirmaniAKBehrensCChenHAhmadianMNowakJAMullerCMinnaJDGazdarAF1997 Deletions of chromosome 3p are frequent and early events in the pathogenesis of uterine cervical carcinoma Cancer Res 57 31543158 9242443

